# HORDB 2.0: a comprehensive dataset of peptide hormones

**DOI:** 10.1038/s41597-026-07514-7

**Published:** 2026-05-29

**Authors:** Chenxin Hu, Yuye Ran, Xingzhen Lao, Heng Zheng

**Affiliations:** China Pharmaceutical University, Nanjing, China

## Abstract

Peptide hormones are key signaling molecules secreted by endocrine cells that regulate vital physiological processes such as metabolism, growth, development, and reproduction. Advances in protein detection and computational approaches have led to the continuous identification of novel peptide hormones, highlighting their potential in basic research, clinical applications, and drug development. To support systematic studies in this field, we present HORDB 2.0, an open-access, manually curated dataset of peptide hormones. This dataset comprises 7,390 entries, including 7,307 peptide hormones and 83 peptide hormone drugs, corresponding to 372 marketed formulations. The dataset includes annotations on sequence, activity, structure, physicochemical properties, patents, clinical data, and literature references, where available. Additional parameters, including IC₅₀, EC₅₀, ED₅₀, and blood–brain barrier permeability, are also provided to facilitate peptide modification and drug design. HORDB 2.0 represents an expanded and enriched resource for peptide hormone research and therapeutic development, and may support downstream applications in endocrinology, pharmacology, and peptide-based drug discovery.

## Background & Summary

In 1922, the extraction of insulin from animal pancreases for diabetes treatment marked the introduction of peptides as therapeutic agents^1^. Peptides represent a unique class of therapeutics, occupying an intermediate molecular space between small molecules and proteins^2^. This unique position enables them to target protein surfaces often inaccessible to conventional small-molecule drugs. Over recent decades, interest in peptide-based clinical therapies has grown substantially. Their unique structural properties allow them to target protein–protein interfaces that are often inaccessible to conventional small molecules. Over recent decades, peptide drugs have gained increasing attention for their high selectivity, low off-target toxicity, favorable safety profiles, and cost-effective production^3^. With continued advances in peptide synthesis, modification, and analytical technologies, together with progress in drug delivery systems and emerging computational approaches such as artificial intelligence and big-data-driven models, peptide drug research and development has entered a broader stage^4^. Consequently, peptide therapeutics are being developed for diverse indications, including metabolic disorders, infectious diseases, chronic pain, and cancer, with more than 80 peptide drugs already approved worldwide, and more than 200 additional peptide drugs currently under clinical investigation^2,3,5^.

Peptide hormones, generally composed of fewer than 100 amino acids, are secreted by specialized endocrine cells into the bloodstream, where they act as essential signaling molecules by binding to membrane-bound receptors^6^. Their pivotal biological functions have made them promising candidates for therapeutic development. Hormonal peptides, as therapeutic agents, are involved in a wide range of clinical applications, including diabetes, obesity, cancer, cardiovascular and cerebrovascular diseases, gastrointestinal disorders, endocrine dysfunctions, and neurological diseases^7–11^. For example, semaglutide is a subcutaneous GLP-1 receptor agonist, an artificially synthesized and highly optimized hormonal peptide that mimics the effect of natural GLP-1 in the human body, but with stronger efficacy and a longer duration of action^7,12^. It was approved for the treatment of obesity in 2021, and accumulating evidence suggests additional cardioprotective effects^13^. Octreotide is a well-established hormonal peptide drug that plays a pivotal role in the treatment of neuroendocrine tumors and various other disorders^14^. It mimics endogenous somatostatin and exhibits greater potency and an extended half-life, thereby improving its suitability for clinical application^15^.

The growing volume of peptide-related research highlights the need for systematic and structured data organization. Several peptide-related resources have been developed to address this demand; however, many still have notable limitations. DRAMP 4.0 is a manually curated, open-access dataset focusing on antimicrobial peptides and provides detailed annotations, including information related to cytotoxicity^16–18^. DCTPep is a comprehensive resource dedicated to cancer therapy peptides and includes not only conventional anticancer peptides but also peptides associated with broader aspects of cancer treatment^19,20^. In addition, some resources focus specifically on peptide hormones. Hmrbase2 contains information on approximately 2,000 hormones and 3,000 receptors, but its coverage of peptide hormones remains incomplete and its updates are relatively limited^21^. Similarly, AHD 2.0 mainly focuses on *Arabidopsis thaliana* hormone-related information, with a narrower scope and more limited peptide-centered annotations^22^.

To address these gaps, we previously developed HORDB, a curated resource dedicated to peptide hormones, and have now expanded it into HORDB 2.0. Compared with the previous version, HORDB 2.0 shows substantial improvements in both data volume and annotation depth. The total number of peptide hormone entries has increased by approximately 27.5%, from 5,729 to 7,307, while peptide hormone drug records have increased from 40 to 83. Corresponding marketed formulation records have also increased from 255 to 372. In addition, the dataset now incorporates 1,916 three-dimensional structural files, representing a marked increase from the previous version, including both experimentally resolved structures and AI-predicted models. New annotation fields, such as disease associations, Gene Ontology functional terms, glycosylation and mutagenesis information, and pharmacokinetic parameters including Kd and half-life, have also been incorporated to improve the utility of the dataset for both basic research and therapeutic development.

HORDB 2.0 expands taxonomic coverage to include peptides derived from humans, animals, and plants, and is organized into two main parts: peptide hormones and peptide hormone drugs, together with their corresponding marketed formulations. Each peptide hormone entry is annotated with sequence, family classification, biological source, post-translational modifications, structural information, functional description, and supporting references. Drug-related entries further include information such as therapeutic indications, active ingredients, developer affiliations, approval dates, and routes of administration. As a curated and publicly available dataset, HORDB 2.0 provides a comprehensive collection of peptide hormone-related information and may serve as a useful resource for peptide hormone research, endocrinology, pharmacology, and peptide-based therapeutic discovery.

## Methods

### Data collection and processing

Peptides included in HORDB 2.0 met the following criteria: (i) fully elucidated amino acid sequences; (ii) sequence length of fewer than 100 amino acids; and (iii) mature peptide forms, with precursor and signal peptide regions removed where applicable. The collected data were organized into two categories: peptide hormone information and peptide hormone drug information.

**Fig. 1:**
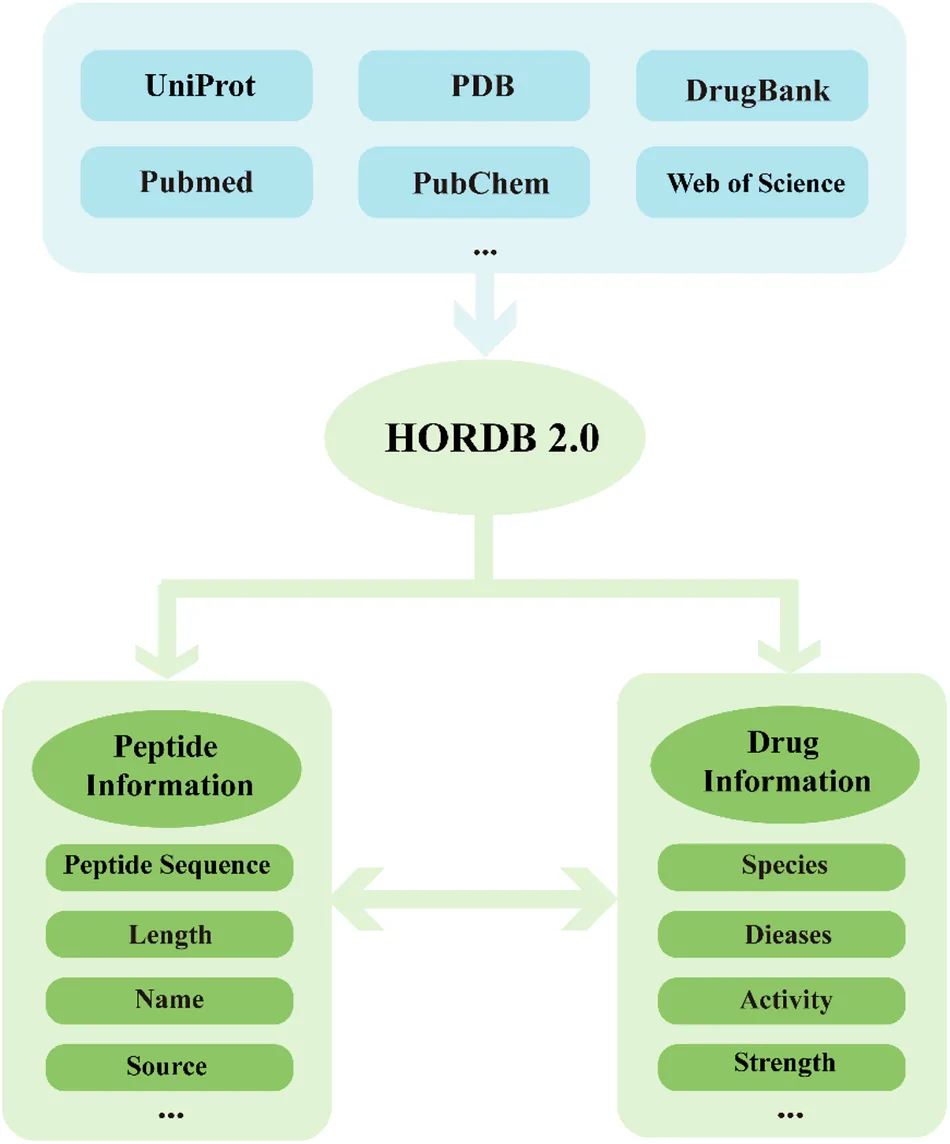
The process of dataset construction.

Peptide hormone data were obtained primarily from UniProt^23^, the Protein Data Bank (PDB)^24^, the AlphaFold Protein Structure Database^25^, and relevant literature retrieved through PubMed (https://pubmed.ncbi.nlm.nih.gov/). For peptide hormone drug data, information on active ingredients, indications, approval status, and marketed formulations was manually curated from published scientific literature, the Drugs@FDA resource (https://www.accessdata.fda.gov/scripts/cder/daf/), and European Medicines Agency public assessment documents, including European Public Assessment Reports (EPARs) (https://www.ema.europa.eu/en/medicines/what-we-publish-medicines-when/european-public-assessment-reports-background-context). To improve accuracy and facilitate cross-referencing, curated drug entries were further checked against corresponding records in DrugBank^26^ and PubChem^27^. Sequences and three-dimensional structural information were collected from the aforementioned public resources using targeted keyword searches, including “hormone” and “peptide hormone”. For peptide entries with available structural information, experimentally resolved structures and publicly available predicted models were recorded where applicable. The physicochemical parameters of each peptide, including molecular weight and isoelectric point, were calculated using the ExPASy ProtParam tool^28^. The overall workflow for data collection, curation, and integration is summarized in Fig. 1.

### Data licensing and redistribution compliance

All primary data integrated into HORDB 2.0 were obtained from publicly accessible resources whose terms of use permit academic reuse and redistribution in accordance with relevant data-sharing policies. Peptide hormone sequence and structure-related information were collected from open-access resources, including UniProt^23^, the Protein Data Bank (PDB)^24^, and the AlphaFold Protein Structure Database^25^, which allow the reuse of associated records and derived datasets for research purposes.

Drug-related information was manually compiled from published scientific literature and official public documents, including publicly available drug-label and regulatory information. Where applicable, curated entries were cross-referenced with external resources such as DrugBank^26^ and PubChem^27^ to improve data traceability and facilitate linkage to source records. In HORDB 2.0, identifiers from these external resources, including DrugBank accession numbers and PubChem compound IDs (CIDs), are provided for cross-referencing and scholarly citation only.

The dataset deposited in Zenodo represents a manually curated and integrated release of peptide hormone-related information and does not constitute bulk redistribution of proprietary source dataset contents^29^. Users are encouraged to consult the original source resources for the most authoritative and up-to-date records, and to follow the licensing terms and usage conditions specified by the respective data providers when reusing the original source data.

## Data Records

The static version of the HORDB 2.0 dataset described in this manuscript has been deposited in Zenodo as record 18530505, with 10.5281/zenodo.18530505^29^. The dataset is organized into a structured archive to facilitate independent reuse and analysis.

Upon download, users will find the following core files:

1. **Core Annotation and Property Files**

- peptide_hormones_information.xlsx
  - **Format:** Microsoft Excel (.xlsx)
  - **Description:** The central data table containing all manually curated peptide hormone entries. Each row corresponds to one unique peptide hormone, with annotations grouped into logical sections.
  - **Key Variables (Column Headers) by Section:**
    - **General Information:** ID (HORDB primary key), UniProt ID, Protein name, Gene name, Organism, Family, Expression, Source, Disease, Comments, Taxonomy, GO Molecular Function, GO Biological Process, GO Cellular Component.
    - **Sequence Information:** Sequence, Length, Propeptide, Signal peptide, Modification, Glycosylation, Mutagenesis.
    - **Activity:** Function, Mechanism, Cross BBB (Blood-Brain Barrier permeability), Target, Target UniProt ID, EC50, ED50, IC50, Kd, Half Life, Link for Activity.
    - **Structure:** Disulfide Bond, PDB_ID/AlphaFold_DB_ID.
    - **Reference:** PubMed ID, Title.
- peptide_physical_information.xlsx
  - **Format:** Microsoft Excel (.xlsx)
  - **Description:** Contains computationally derived physicochemical and amino acid composition properties for each entry in the core data file. The ID column links to peptide_hormones_information.xlsx.
  - **Key Variables:** ID, mass (molecular weight), Formula, pI (isoelectric point), Hydrophobicity, Boman_in (Boman index), Half-Life_m, Aliphatic_in, Instability_in, Extinction_cc (extinction coefficient), Ab280 (absorbance at 280 nm). The file also includes columns for the count or frequency of each standard amino acid (Alanine, Arginine,…, Valine), as well as summary columns for Absent_aa, Common_aa, Basic_r, Acidic_r, Polar_r, and Hydrophobic_r.
- peptide_hormone_drugs_information.xlsx
  - **Format:** Microsoft Excel (.xlsx)
  - **Description:** Records for approved peptide hormone drug entities (Active Pharmaceutical Ingredients, APIs). Information was manually curated from literature and official sources.
  - **Key Variables:** ID (Drug ID), AI (Active Ingredient name), DRUGBANK Accession Number (**for cross-referencing only**), syn (synonyms), Sequence, Sequence Length, Type (e.g., native, analog), Description (therapeutic description), CAS, SMILES, disease (indication), name (drug name).
- marketed_pharmaceutical_preparations_information.xlsx
  - **Format:** Microsoft Excel (.xlsx)
  - **Description:** Details of specific commercial formulations corresponding to the drug entities.
  - **Key Variables:** ID (Formulation ID), ActiveIngredients, DRUGBANK Number (**for cross-referencing only**), Synonyms, ActiveSequence, SequenceLength, Type, Description, CAS, SMILES, Disease, DrugName, Strength, ApprovalYear, Company, MarketingStatus, DosageFormRoute, name. The ActiveIngredients field links this table to peptide_hormone_drugs_information.xlsx.

2. Structure Data Files

- peptide_structure.zip
  - **Format:** Compressed archive (.zip)
  - **Description:** Contains all three-dimensional structural data files in PDB format. The archive includes three subdirectories:
    1. Experimental_PDB/: Contains 72.pdb files of experimentally determined structures. File names correspond to the PDB_ID listed in peptide_hormones_information.xlsx.
    2. AlphaFoldDB_Models/: Contains 851.pdb files of pre-computed models from the AlphaFold Protein Structure Database. File names correspond to the AlphaFold_DB_ID (typically a UniProt ID) listed in the core file.
    3. AlphaFold2_Predictions/: Contains 993.pdb files of structures predicted for this project using AlphaFold 2. File names follow the format [HORDB_ID]_AF2.pdb, where [HORDB_ID] matches the ID in peptide_hormones_information.xlsx.

## Technical Validation

The core data in HORDB 2.0 were manually curated and verified through multiple quality-control procedures. For peptide hormone entries, records collected from public resources and the literature were cross-checked for consistency, and data accuracy was further assessed through random manual sampling. To facilitate data organization and validation, the curated records were structured into four principal categories: peptide information, active peptides, peptide physicochemical properties, and marketed formulations.

For peptide hormone entries, sequence information, source annotations, functional descriptions, structural records, and literature references were manually checked during curation. For peptide hormone drug entries, information on active ingredients, indications, and marketed formulations was cross-verified using published literature, official public documents, and linked external resource identifiers where applicable. Physicochemical properties were generated using a standardized calculation procedure, and structural records were linked to corresponding identifiers to ensure consistency between annotation files and structure files.

In addition, selected records were manually inspected to further evaluate annotation accuracy and internal consistency. Major revisions of the dataset were managed through version control to improve traceability and maintain the consistency of the released dataset.

## Usage Notes

The HORDB 2.0 dataset provides a comprehensive, manually curated collection of peptide hormones and related drugs to support research in endocrinology, pharmacology, and peptide-based drug discovery. The static dataset described in this manuscript is permanently available in Zenodo under the 10.5281/zenodo.18530505^29^. Below, we outline the main downstream applications of the dataset and provide practical guidance for its use.

### Potential Applications

1. **Drug Discovery and Design:** The dataset is a valuable resource for identifying novel peptide hormone leads and optimizing existing ones. The inclusion of sequences, structures, activity parameters (IC₅₀, EC₅₀, Kd), physicochemical properties, and blood-brain barrier permeability annotations enables structure-activity relationship (SAR) studies, *in silico* screening, and rational drug design.
2. **Computational Biology and Modeling:** Researchers can leverage the 1,916 structural files (including experimental and AlphaFold-predicted models) for molecular docking, molecular dynamics simulations, and protein-peptide interaction studies. The standardized sequence and annotation files are suitable for training machine learning models to predict hormone activity, stability, or other bioactive properties.
3. **Comparative and Evolutionary Analysis:** The taxonomic breadth of the dataset (covering human, animal, and plant peptides) allows for comparative genomic and proteomic studies. Users can investigate the conservation, variation, and evolution of peptide hormone families across different species.
4. **Clinical and Translational Research:** The linkage between peptides, their associated diseases, and approved drug information facilitates translational research. It aids in understanding the therapeutic landscape, repurposing existing hormones for new indications, and analyzing the molecular basis of endocrine disorders.

### Practical guidance for data use

The dataset is organized into several core files in the Zenodo repository to facilitate reuse, and detailed descriptions of file contents, formats, and variables are provided in the Data Records section. Users may select different files according to their research aims: for example, peptide_hormones_information.xlsx contains the main curated annotations for peptide hormone entries, peptide_physical_information.xlsx provides calculated physicochemical properties, peptide_hormone_drugs_information.xlsx and marketed_pharmaceutical_preparations_information.xlsx contain drug-related information, and peptide_structure.zip provides structural data for downstream computational analyses. Because HORDB 2.0 integrates information from multiple public resources and published literature, some annotation fields may be unavailable for a subset of entries, and the completeness and consistency of specific variables may vary across peptide and drug records. In addition, some activity-, structure-, and drug-related annotations are derived from heterogeneous sources with different reporting standards. Users are therefore encouraged to interpret individual fields together with the original references and linked source identifiers where appropriate. As the Zenodo-deposited dataset represents a static release, it does not include subsequent updates to the online resource.

### Comparison with the previous version

HORDB 2.0 represents a substantial expansion and enhancement over its predecessor, HORDB 1.0, in terms of data content and annotation depth. HORDB 2.0 includes over 1,900 additional entries and a markedly increased number of structural records, providing a richer resource for peptide hormone research. New annotation fields, such as Gene Ontology terms, disease associations, glycosylation and mutagenesis information, and pharmacokinetic parameters including Kd and half-life, provide broader biological and clinical context. In addition, peptide hormone drugs and marketed formulations are organized into two dedicated sections, which improves the clarity of the dataset structure. The inclusion of nearly 2,000 structural files, including both experimentally resolved and AI-predicted models, further supports structure-based studies and downstream computational analyses. A summary of the main differences between HORDB 1.0 and HORDB 2.0 is provided in Table 1.

**Table 1:** Comparison of HORDB 1.0 and HORDB 2.0.

Aspect	HORDB 1.0	HORDB 2.0
Total entries	6,024	7,390
Peptide hormones	5,729	7,307
Peptide hormone drugs	40	83
Marketed formulations	255	372
3D structural data	408 structures (PDB: 54, AlphaFold: 354)	1,916 structures (PDB: 72, AlphaFold: 851 + 993 predicted)
Information fields per entry	6 categories	Enhanced with disease associations, GO annotations (MF/BP/CC), glycosylation, mutagenesis, Kd, and half-life
Drug information organization	Combined into general entries	Split into two dedicated sections: peptide hormone drugs and marketed formulations
Taxonomic coverage	More limited	Expanded to include peptides from humans, animals, and plants

## Data Availability

The HORDB 2.0 dataset generated and described in this study—including all annotated peptide hormone entries, physicochemical properties, drug information, associated sequences, and structural files—has been deposited as a static, versioned archive in the Zenodo repository under the accession code 10.5281/zenodo.18530505. This archive contains the complete dataset in machine-readable formats (.xlsx, .fasta, .pdb, .zip) as detailed in the Data Records section. All data are freely available for download and reuse under the terms specified in the repository.

## Code availability

No custom code is associated with this Data Descriptor.
